# Investigators are human too: outcome bias and perceptions of individual culpability in patient safety incident investigations

**DOI:** 10.1136/bmjqs-2024-017926

**Published:** 2025-02-10

**Authors:** William Lea, Luke Budworth, Jane O'Hara, Charles Vincent, Rebecca Lawton

**Affiliations:** 1 University of Leeds, Leeds, UK; 2 York & Scarborough Teaching Hospital NHS Foundation Trust, York, UK; 3 Bradford Institute for Health Research Yorkshire Quality and Safety Research Group, Bradford, UK; 4 THIS Institute, Cambridge, Cambridgeshire, UK; 5 Department of Experimental Psychology, University of Oxford, Oxford, UK; 6 University of Leeds School of Psychology, Leeds, Leeds, UK

**Keywords:** Patient Safety, Cognitive biases, Health policy

## Abstract

**Background:**

Healthcare patient safety investigations inappropriately focus on individual culpability and the target of recommendations is often on the behaviours of individuals, rather than addressing latent failures of the system. The aim of this study was to explore whether outcome bias might provide some explanation for this. Outcome bias occurs when the ultimate outcome of a past event is given excessive weight, in comparison to other information, when judging the preceding actions or decisions.

**Methods:**

We conducted a survey in which participants were each presented with three incident scenarios, followed by the findings of an investigation. The scenarios remained the same, but the patient outcome was manipulated. Participants were recruited via social media and we examined three groups (general public, healthcare staff and experts) and those with previous incident involvement. Participants were asked about staff responsibility, avoidability, importance of investigating and to select up to five recommendations to prevent recurrence. Summary statistics and multilevel modelling were used to examine the association between patient outcome and the above measures.

**Results:**

212 participants completed the online survey. Worsening patient outcome was associated with increased judgements of staff responsibility for causing the incident as well as greater motivation to investigate. More participants selected punitive recommendations when patient outcome was worse. While avoidability did not appear to be associated with patient outcome, ratings were high suggesting participants always considered incidents to be highly avoidable. Those with patient safety expertise demonstrated these associations but to a lesser extent, when compared with other participants. We discuss important comparisons between the participant groups as well as those with previous incident involvement, as victim or staff member.

**Interpretation:**

Outcome bias has a significant impact on judgements following incidents and investigations and may contribute to the continued focus on individual culpability and individual focused recommendations observed following investigations.

**WHAT IS ALREADY KNOWN ON THIS TOPIC**

- Judgements of individual responsibility are often influenced by knowledge of outcomes for victims, a phenomenon known as outcome bias. In healthcare, this bias could lead to disproportionate blame placed on individuals involved in patient safety incidents rather than a focus on systemic issues.

**WHAT THIS STUDY ADDS**

- We found that when patient outcomes were worse following a patient safety incident, greater responsibility was assigned to healthcare staff involved, there was a stronger motivation to investigate, and an increased likelihood of punitive recommendations. Expertise in patient safety may reduce, but does not eliminate, these biases.

**HOW THIS STUDY MIGHT AFFECT RESEARCH, PRACTICE OR POLICY**

- Research, practice and policy should focus on understanding and managing cognitive biases among investigators. Enhancing training and oversight in this area could improve the effectiveness and fairness of safety investigations.

## Introduction

 Globally, efforts to improve patient safety have relied heavily on the retrospective investigation of patient safety incidents.^1^ This approach is founded on an interpretation of safety theory, which proposes that errors are multifactorial in nature and that identifying and addressing organisational latent failures through investigation and generating recommendations will reduce future recurrence.^2 3^

Recent publications^4^,^13^ including our own review^14^ have identified that the overwhelming majority of recommendations developed following serious incident investigations would be categorised as ‘weak’ according to the framework developed by Hibbert and colleagues.^12^ Rather than addressing latent failures of the system (eg, design of equipment), the target of recommendations is most often on the behaviours of individuals, such as reminders, writing or rewriting policies and (re-)training staff. The patient safety movement has struggled to shift the focus from people to systems; and this may be a reason why we are still not ‘learning’ from patient safety investigations and therefore not reaping the benefits of careful analysis.^11 15 16^ In fact, it is now acknowledged that investigations can, themselves, compound or add harm to those involved or affected by the incident, investigation or subsequent recommendations.^17^ To address these problems, we need to better understand the flaws in the incident investigation process itself.

There has long been evidence that our judgements (attributions) of individual responsibility or culpability are driven by the outcome of an accident or adverse event.^18 19^ In other words, the same behaviour (parking on a slope without putting the handbrake on) is judged more harshly when the outcome is bad (the car moves and runs someone over), than when the outcome is not (the car does not move or moves but no-one is harmed). While the original studies of this bias were conducted within the field of road traffic accidents or legal settings, subsequent studies in healthcare have demonstrated that our judgements, of staff actions and behaviours, are also influenced by what is known as ‘outcome bias’.^20^,^23^ Outcome bias involves evaluating an individual or procedure responsible for an outcome; the evaluation is considered biased when outcome information is given excessive weight.^24 25^ The above studies highlight an important issue that has largely been ignored within the policy and practice of healthcare incident investigation—that those investigating, or even consulted as part of an investigation, are potentially influenced by psychological biases—they are human too. Outside of healthcare, it is suggested that the impact of bias is broad, effecting what information is collected and how the analysis is carried out, by and with whom.^26^ Bias has the potential to cause an inappropriate focus on individual culpability, and narrow or skew the exploration and understanding of an incident’s causation.^27^ The impact of cognitive biases is further complicated by commonly held fallacies about their nature, for instance, that they only affect corrupt, malicious or incompetent individuals, that experts are ‘immune’, and that simply being aware of biases allows individuals to overcome their affect.^26^

The purpose of this study was to examine the impact of outcome bias on the judgements and recommendations following hospital-based incident investigations. We also explore whether these biases might be reduced or eliminated through training or expertise in patient safety. We test the following hypotheses:

**Hypothesis 1:** increasing outcome severity is associated with increased judgements of responsibility and avoidability.

**Hypothesis 2:** increasing outcome severity is associated with increased judgements of the importance of investigation.

**Hypothesis 3:** increasing outcome severity is associated with more recommendations, and more punitive recommendations.

**Hypothesis 4**: expertise in patient safety will reduce outcome bias.

## Methods

### Study design

**Figure 1:**
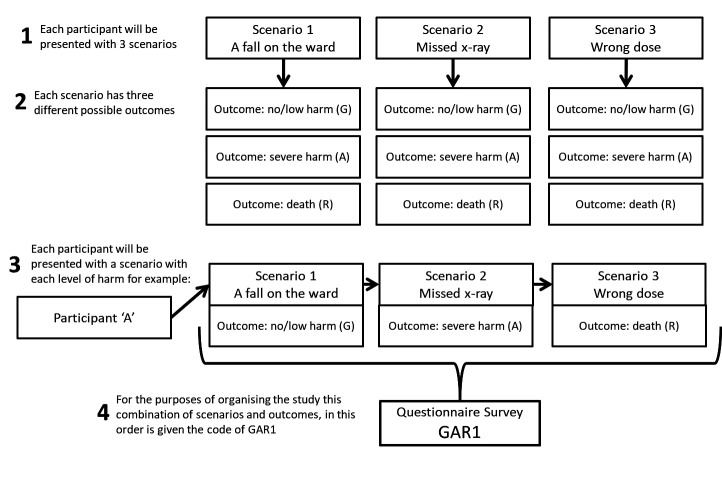
Participant scenario allocation. (G = no/low harm, A = severe harm, R = death)

Using an experimental design, we developed and distributed an online questionnaire presenting three fictitious incident scenarios, along with the findings of an investigation for each (see online supplemental appendix 1 for the scenarios and online supplemental appendix 2 for an example questionnaire). The scenarios were based on real incidents and produced by WL, JOH, RL and CV who have a combined 86 years’ experience in patient safety research, systematic review of patient safety incidents and analysis of incident investigations and recommendations. While the scenarios remained the same, regarding the events leading up to the incident and the contributing factors identified by an investigation, across three conditions, we manipulated the outcomes for the patient ((1) no/low harm, (2) severe harm, (3) death). Participants were presented with all three scenarios; one resulting in no harm, one severe harm and one that resulted in death (figure 1). The order in which scenarios were presented to participants was randomised, resulting in nine versions of the questionnaire, to mitigate order effects.^28 29^ Repeated measures within individuals were intentionally designed to enhance the statistical power of the study, compared with a purely between-subject design. By presenting each participant with all three outcome scenarios, we control for individual differences, thus reducing variability and increasing the precision of our estimates. This approach allows us to detect smaller effects with a given sample size, as each participant serves as their own control.

For each incident scenario, participants were asked to rate, on a 1–5 Likert scale, how responsible the involved healthcare professionals were in causing the incident (1=not at all, 5=entirely), how avoidable it was and how important an investigation of the incident was. Participants were also asked to select up to five (of a possible eight) recommendations to prevent incident recurrence. To verify that our manipulation of the outcomes was effective, we asked participants to identify the outcome of each scenario (no/low harm, severe harm or death) as they perceived it.

### Participants

Participants were recruited via adverts shared on the social media platform Twitter/X. A link in the advert directed potential participants to the online questionnaire (online supplemental appendix 2). The first section of the questionnaire contained questions to establish suitability for the study, which participant group they belonged to, and to gain consent. The remainder of the questionnaire presented participants with scenarios 1–3, in a random order. Following each scenario, participants were asked to answer questions as detailed above. While participants were not given the option to save progress and complete at a later time, there was no time limit on how long participants could take to complete the questionnaire. As no name or contact information was collected from participants, there was no follow-up for completion.

Participants were recruited from three groups: (1) public, (2) healthcare staff, (3) people with expertise in patient safety or investigation of patient safety incidents.

The group of people with expertise in patient safety/investigations were further divided into those who had a clinical background (clinical experts), and those from a non-clinical background, such as researchers, policymakers or human factor engineers (non-clinical experts). Clinical expertise was defined as someone who had a background of working in healthcare (eg, nurse, doctor, manager) and having gone on to complete at least 10 investigations, undergone patient safety or investigation training, and held a job role involving patient safety or investigation. Non-clinical expertise was defined as practical or academic job role involving patient safety or human factors in relation to incident investigation. WL and RL independently reviewed participants’ answers about professional background, training and involvement in investigations, in order to assign to an expertise category. WL and RL compared allocations, any unresolved disagreements, were discussed with a third author (JOH).

### Sample and setting

Given the innovative nature of this research, precise effect size estimates were unavailable, complicating sample size calculation.^30 31^ However, drawing from similar studies,^21^,^23^ we conducted a power analysis considering plausible effect sizes (d=0.4) and variability. This analysis indicated that a minimum sample size of 150 would ensure sufficient statistical power and reliable results.

### Analysis of recommendation choice

Following each scenario, participants were presented with eight recommendations; two punitive, followed by two weak, two medium and two strong, as defined by the action hierarchy.^12 14 32^ The recommendations were drafted by WL and reviewed and modified by JOH, RL and CV (see online supplemental appendix 1 for recommendations). As well as descriptive statistics for participant recommendation choices, a weighted recommendation score was used to produce a single number representing a participants’ recommendation choice. The recommendation score (RecScore) was calculated to represent degree of ‘system-orientated’ versus ‘individual-oriented’ recommendation selections. The minimum possible score was −1, representing a choice of recommendations that could be considered punitive, and the maximum possible score was 3, representing a system-focussed selection. RecScore was calculated as below:


$$RecScore=\frac{\left(\left(n\;punitive\times -1\right)+\left(n\;weak\times 1\right)+\left(n\;of\;medium\times 2\right)+\left(n\;strong\times 3\right)\right)}{Total\;number\;of\;recommendations\;selected}$$

### Statistical analysis

In order to avoid assumptions about the equidistance of points in the Likert-Scale responses (responsibility, avoidability, importance of investigation), we produced both ordinal logistic regression models (results available on request) and multilevel linear models. Given the outcomes were similar, we opted to report the results of the linear models for simplicity.

Given the nested design (repeated measures within individuals), multivariable linear mixed (multilevel) models (MLM) were used (fit via *Lmer* in R), with random intercepts specified for participants.^33 34^ We ran separate models for each outcome, namely, responsibility, avoidability, importance of investigating, number of recommendations (nRec) and recommendation score (RecScore).

There is evidence to suggest that age and gender, which might alter cognition and attitudes, could be important confounding factors^35^,^39^ as well as the differences in the scenario ‘story’, and participant group. We also felt it reasonable to consider participants’ previous involvement in incidents as a potential confounder, as a victim, staff member involved or both.

For each outcome, we built three models with an increasing number of variables to account for these potential confounding factors:

- *Model 1*—scenario outcome, participant age, participant gender.
- *Model 2*—model 1 variables+scenario, participant group.
- *Model 3*—model 2 variables+participant previous incident involvement (none/victim/staff member).

As well as coefficient estimates, several statistics were produced from the models to evaluate model fit. These included (1) the intraclass correlation coefficient (ICC), providing an estimate of the proportion of total variance in each outcome that was attributed to the grouping structure in the data (ie, between participant differences), (2) R^2^_Marginal_, representing the proportion of variance explained solely by the fixed effects, disregarding the random effects: gauging how well predictors elucidate the outcome variable, excluding the multilevel structure’s consideration and (3) R^2^_Conditional_, representing the same as R^2^_Marginal_ but also including the variance explained by the random effects.

We also produced Bayesian Information Criterion (BIC) statistics, which allowed us to compare model fit between models specifying the same outcome (lower scores=better fit). Primarily these were used to gauge whether adding further predictors in model 2 or 3 improved model fit.

Subgroup analysis was performed to calculate mean differences in responsibility ratings, nRec and RecScore between the participant groups.

The correlation between designed scenario outcome and participant reported outcome was calculated to check the manipulation.

Throughout the manuscript, we specified alpha at 5% (two tailed).

## Results

Two hundred and twelve participants completed the questionnaire (table 1), resulting in 636 observations (three observations per participant). Missing data were low, 41 of 5936 data items (0.69%). Participants were mostly women (n=166, 78.3%) and had an average age of 44 (range 17–80, SD=13.6). Members of the public made up the largest group (n=100, 47.2%), followed by healthcare staff (n=71, 33.5%) and experts (n=41, 19.3%; clinical 30; researcher 11). Approximately a third of participants had been a victim of a safety incident (either personally or a close relative) (n=63, 30.0%), or involved as a member of staff (n=70, 33.0%). A small proportion of participants had been both a victim and a member of staff involved in a safety incident (n=19, 9.0%).

**Table 1:** Participant characteristics

	n	%
Public	100	47.2%
Healthcare staff	71	33.5%
Experts	41	19.3%
Female	166	78.3%
Male	45	21.2%
Other/non-binary	1	0.5%
**Previous incident involvement**		
None	98	46.2%
Victim	44	20.8%
Healthcare staff	51	24.1%
Both	19	9.0%
Total	**212**	

### Manipulation check

Designed outcome severity was highly correlated with participant-reported severity (scenario 1: r=0.967, p<0.001, scenario 2: r=0.912, p<0.001, scenario 3: r=0.936, p<0.001).

### Multilevel modelling

When reporting results of MLM below, we refer to those obtained from the model with the lowest BIC, for each outcome. The difference between BIC values across the models was not significantly different and R^2^_c_ values ranged from 40% to 54%. The ICC ranged from 26.8% to 50.9% across models indicating a significant degree of clustering, supporting the use of MLM.

See online supplemental table 1, appendix 3 for full model results. Across the models, there appeared to be no significant effects for the adjustment variables age or gender. There were significant effects for scenario (fall, X-ray, wrong dose) in all outcome variables; in other words, the details of the incident scenarios (eg, events and people involved) had effects on participants’ judgements and responses to all questions. Having been a victim of an incident (personally or close family member) had a significant effect on responsibility ratings ((β=0.50 (CI 0.20 to 0.83), p=<0.001) and importance of investigating ((β=0.31 (CI 0.04 to 0.57), p=<0.001). Having been a staff member involved in an incident before appeared to have no significant effects on the outcome variables.

### Hypothesis 1: increasing outcome severity is associated with increased judgements of responsibility and avoidability

This hypothesis was partly supported. As the outcome for the patient in the scenario became more severe, participants judged the staff involved in the incident as more responsible for causing it. This is demonstrated by the increasing responsibility rating means for no/low harm (2.99), severe harm (3.16) and death (3.25) in table 2. These means are adjusted for age, gender, scenario and participant group. Multilevel modelling demonstrated a significant association between outcome severity and responsibility ratings, the most significant difference noted between death and no/low harm ((β=0.26 (CI 0.11, 0.41), p≤0.001) (table 2, online supplemental table 1, appendix 3).

**Table 2:** Mean response ratings*.* by outcome and participant group, with 95% CIs

	Outcome			Participant group			
	No/low-harm	Severe harm	Death	Public	Staff	Clinical experts	Non-clinical experts
Responsibility	2.99 (2.78,3.20)	3.16 (2.95,3.37)	3.25 (3.04,3.46)	3.67 (3.46,3.88)	3.39 (3.17,3.61)	2.82 (2.48,3.16)	2.66 (2.11,3.2)
Avoidability	4.02 (3.84,4.2)	3.98 (3.8,4.16)	3.98 (3.8,4.16)	4.31 (4.14,4.48)	3.94 (3.76,4.12)	3.78 (3.5,4.06)	3.93 (3.48,4.38)
Importance of investigating	4.09 (3.92,4.27)	4.48 (4.3,4.65)	4.73 (4.56,4.9)	4.43 (4.26,4.6)	4.54 (4.36,4.72)	4.39 (4.11,4.67)	4.37 (3.93,4.82)
Number of recommendations	3.63 (3.4,3.86)	3.56 (3.34,3.79)	3.83 (3.6,4.06)	3.82 (3.59,4.04)	3.92 (3.68,4.16)	3.44 (3.07,3.81)	3.53 (2.94,4.11)
RecScore	2.04 (1.94,2.13)	1.97 (1.87,2.07)	1.96 (1.86,2.06)	1.84 (1.71,1.96)	1.81 (1.71,1.91)	2.07 (1.91,2.24)	2.23 (1.99,2.47)

RecScore, recommendation score.

**Table 3:** Number and types of recommendations selected

	Number of recommendations selected within each category				Total	Average total recommendations selected by each participant
Patient outcome severity	Punitive (1+2)	Weak* (3+4)	Medium* (5+6)	Strong* (7+8)		
Death	67	227	311	246	851	4.01 (SD 1.05)
Severe harm	45	231	295	222	793	3.74 (SD 1.21)
No/low harm	42	224	304	238	808	3.81 (SD 1.14)
Total	154	682	910	706	2452	
	6.3%	27.8%	37.1%	28.8%		

* As defined by the action hierarchy (NPSF 2021).

**Table 4:** Number of participants selecting punitive recommendations by participant group

	Death		Severe harm		No/low harm	
Group	n	% of group	n	% of group	n	% of group
Public	34	34.0%	27	27.0%	25	25.0%
Healthcare staff	18	25.4%	9	12.7%	9	12.7%
Experts	10	24.4%	5	12.2%	6	14.6%

Participants were asked to rate avoidability of the incident. Findings in table 2 and the multilevel model show little difference in the ratings of avoidability for the different outcomes of low harm (4.02), severe harm (3.98) and death (3.98). All ratings were high, suggesting that irrespective of outcome, participants considered these incidents to be highly avoidable.

### Hypothesis 2: increasing outcome severity is associated with increased judgements of importance to investigate

This hypothesis was supported. All ratings were above 4 on the five-point scale. However, when the outcome for the patient was death, the mean score for importance of investigation was 4.73 compared with 4.48 for severe harm and 4.09 for no/low harm. Multilevel modelling (online supplemental table 1, appendix 3) confirmed a statistically significant association between outcome severity and importance of investigating, with the biggest difference observed between no/low-harm and death (β=0.63 (CI 0.50, 0.76), p=<0.001) (table 2, online supplemental table 1, appendix 3).

### Hypothesis 3: increasing outcome severity is associated with selecting more recommendations and more punitive recommendations

Participants were asked to select up to 5 recommendations per incident and 2452 recommendations were selected across 636 incidents. There was no significant observed differences between the nRec selected for no/low harm incidents (average 3.81, SD 1.05), severe harm (3.74, SD 1.21) or death (4.01, SD 1.05). While the models did demonstrate a statistically significant increase in nRec selected for death outcome versus no/low harm outcome, the difference was very small (β=0.20 (CI 0.02, 0.38), p=0.03), representing a fifth of a recommendation (table 3, online supplemental table 1, appendix 3)

Table 3 illustrates the types of recommendations selected, categorised as either punitive (n=154, 6.3%), weak (n=682, 27.8%), medium (n=910, 37.1%) or strong (n=706, 28.8%), in terms of their likelihood of improving safety (see online supplemental appendix 1 for recommendations).^12 14^ It is important to highlight that punitive recommendations made up 8% of those selected when the outcome for the patient was death, 6% for severe harm and 5% for no/lo harm, suggesting that punitive recommendations are more likely to be selected when the outcome for the patient is worse.

Multilevel modelling demonstrated that mean recommendation scores, across participants groups, reduced as the patient outcome became more severe, indicating a more individual-focus to recommendation choices. RecScore was lower when the outcome for the patient was death versus no/low harm and severe harm versus no/low harm, but the differences were not statistically significant ((β=−0.08 (CI −0.16, 0.00, p=0.057) and (β=−0.06 (CI −0.15, 0.02, p=0.137)).

### Hypothesis 4: expertise in patient safety will reduce outcome bias

Our results suggest that those with non-clinical or clinical expertise in safety assign less responsibility to staff for causing an incident than staff (difference=0.73 (95% CI 0.14 to 1.32) and 0.57 (95% CI 0.18 to 0.96)) and the public (difference=1.01 (95% CI 0.44 to 1.58) and 0.85 (95% CI 0.48 to 1.22)); with no significant difference between the public and staff (difference=0.28 (95% CI −0.01 to 0.57))(online supplemental table 2, appendix 3). Furthermore, those experts from a non-clinical background appear to assign less responsibility to staff than those from a clinical background (tables2  4, online supplemental table 2, appendix 3).

We observed no difference in mean avoidability ratings between staff and experts (table 2), but we observed a small difference between both the public and staff (0.37 (95% CI 0.13 to 0.61)) and public and clinical experts (0.53 (95% CI 0.22 to 0.84))(online supplemental table 2, appendix 3). Our results suggest that those with in-depth knowledge of the clinical environment (staff and clinical experts) may perceive incidents as less avoidable than the public or non-clinical experts.

We observed no difference between participant groups in ratings of importance of investigating the incidents within the vignettes, with mean ratings ranging from 4.37 for non-clinical experts to 4.54 for healthcare staff (table 2).

There were no significant differences in the total nRec that participants from different groups selected (table 2 and online supplemental table 2, appendix 3). We did, however, observe differences in the types of recommendations that were selected. Those with patient safety expertise are likely to select stronger recommendations, according to the AH^12 14 29^ than members of the public or healthcare staff, with the biggest differences in recommendation score (scored between −1 and 3) between non-clinical experts and the public (0.35 (95% CI 0.15 to 0.55)) and clinical experts and the public (0.32 (95% CI 0.18 to 0.46))(online supplemental table 2, appendix 3. It is important to highlight the differences observed in the selection of punitive recommendations (table 4). The percentage of public selecting punitive recommendations increased from 24%, at the no/low harm level, to 27% and 34% at severe and death harm levels, respectively. On the other hand, 12.2%–14.6% of staff and experts selected punitive recommendations for no/low harm and severe harm, which increased to 25.4% and 24.4% when the outcome for the patient was death. In other words, more staff and experts selected punitive recommendations when the patient outcome was worse.

## Discussion

The results of this study show that outcome knowledge is associated with changes in how individuals judge and respond to incidents, irrespective of their background (public/professional/expert) or previous experiences with incidents (harmed or involved). While this association has been demonstrated in other domains, to the authors’ knowledge, this is the first study to examine this issue specifically in the context of healthcare incident investigations.

Some of the absolute effects on responses, in our results, appear small. Despite this, we propose even small effects on responses could have a significant impact on patient safety investigations. First, we have demonstrated the effect of outcome bias on three aspects of the investigation process (whether to investigate, the responsibility of staff and the selection of recommendations). Rather than considering the impact of each individually, we need to consider the cumulative impact of outcome bias that may occur at repeated time points, on multiple decisions and the many people involved through the course of a single investigation.^26^ Second, given 2–3 million incidents are reported in England alone even a very small impact on each incident investigation has far-reaching consequences at a national level.

### Increasing outcome severity is associated with increased judgements of responsibility but not avoidability

Our results suggest that the more severe the outcome of an incident, the greater the responsibility that will be assigned to the staff involved. This effect of outcome-knowledge bias on responsibility has been demonstrated many times in different contexts,^18 19^ and so it is not surprising to find it within the context of healthcare incident investigations. Thus, while Dekker and others highlight the need for a shift in responsibility for patient safety from human error to symptom of trouble within a system,^40^ unrecognised outcome bias may be hindering progress in this direction.

While avoidability did not appear to be associated with worsening outcome severity, our results suggest participants considered all the incidents relatively avoidable, with mean ratings across all outcome severities and group categories being approximately 4 out of 5. These generally high scores may be the result of hindsight bias where once the outcome is known people tend to alter their perception of how likely an event was to occur, sometimes referred to as the ‘knew-it-all-along’ effect.^41^ While both hindsight bias and outcome bias involve the projection of new information into the evaluation of past events or actions, hindsight bias involves the denial that outcome information has influenced judgements.^42^,^44^ As we did not ask participants how they came to their judgements, we have not specifically examined hindsight bias.

Hindsight and outcome biases may cause investigators to focus on poor decisions or missed opportunities rather than other factors, in incident causation.^40 45 46^ The avoidability or preventability of an incident may be described as complex (Meyer 2023) and beyond the scope of this paper.^47^

### Increasing outcome severity is associated with increased judgements of importance to investigate

Our results suggest people consider it more important to investigate an incident when a patient comes to greater harm. There are a number of purported reasons for carrying out investigations: to improve the safety and quality of care; to assign accountability; to litigation or compensation, for restoration, or to repair or protect organisational reputation.

This study was designed, so that the events leading up to the incident and the systems in which the incidents occurred were identical; it was by chance that each of the alternative outcomes occurred (no/low harm, severed harm and death). Therefore, it could be argued that the ‘opportunities for learning’ were the same, no matter the outcome for the patient. When allocating resources for investigation, organisations are encouraging more proportionate responses and a move away from responses based on subjective thresholds and definitions of harm.^48^ Our study demonstrates that level of harm remains an important factor in how people decide how to investigate incidents. New policies and frameworks alone might struggle to overcome this especially in jurisdictions that mandate investigations for higher harm incidents, thus legitimising outcome bias in health policy.^49 50^

### Increasing outcome severity is not associated with more recommendations but is associated with more punitive recommendations

While there was no observed difference in the number of recommendations selected between levels of harm or participant groups, there was an overwhelming tendency to select recommendations than not (an average of 4 out of 5 were selected for all three scenarios). Adams *et al* demonstrated that humans prefer additive change than subtractive, for example, adding a checklist rather than removing one.^51^ While we cannot be sure this is the case in our study, our results imply that safety investigations could contribute to the creation of safety clutter or low-value safety practices.^52 53^

Our results suggest that knowledge of a severe patient outcome, alone, may increase the chance of punitive recommendations being selected. This inappropriate focus on individuals may distract improvement efforts away from a system approach, such as redesigning processes and equipment. The impact of outcome bias also has the potential to contribute to the already present culture of blame within healthcare, hampering efforts to improve patient safety and encouraging the adoption of potentially harmful defensive practice among clinicians.^54 55^

### Expertise in patient safety can reduce some biases

Our results demonstrate differences in how people from different backgrounds and expertise respond to incidents. Expertise appears to mitigate perceptions of responsibility and the reduce the selection of punitive recommendations but not fully. When the patient outcome was death, the proportion of experts and staff selecting punitive recommendations doubled. This suggests that clinical knowledge or patient safety expertise alone, may be useful but insufficient to mitigate against outcome bias. This is perhaps expected given research showing that a number of factors affect the presence or impact of biases, such as expertise, previous experience, cognitive ability, bias awareness, tolerance of ambiguity and organisational culture.^46 56 57^ The interesting differences observed between clinical and non-clinical experts suggest that the type and origin of expertise are important in ensuring effective investigations. Further research should focus on understanding the impact of investigator cognition and bias as well as other factors such as professional background and training on investigations.

### Implications for policy and practice

We highlight the different and sometimes conflicting responses of the public, staff and experts, which will need to be considered within policy and future research. While patient harm plays an important part in individuals’ responses to an incident, policymakers will need to consider when harm severity is justification for investigation and when it is not. Those with responsibility for oversight of investigations and investigators should have an understanding of bias and how it might impact investigations. Future research should continue to develop and empirically test strategies to mitigate the impact of cognitive biases on investigations, such as investigator training, blinding and unmasking (eg, to patient outcome) and independent verification.^46^ Further research is needed to understand not only the impact of bias on investigation but also the increasing number of alternatives to traditional investigation in patient safety, such as After-Action Review, 'SWARM' and Structured Judgement Review.^58 59^ Policymakers and organisations should ensure that investigations are led by those with expertise and experience and would benefit from defined competencies for investigators.

### Limitations

Our study has a number of limitations. We examined outcome bias in individuals; but investigations may be carried out by a team; future research is needed to understand how investigations performed by a group may or may not mitigate the impacts of outcome bias, or indeed introduce other cognitive and social issues, such as ‘Group Think’ or shared information bias, on the entire life cycle of an investigation.^60^,^62^ While a team of investigators is recommended, in our experience and in discussion with a wide range of stakeholders, we suggest that not all investigations are carried out by teams. The most ‘serious’ incidents might well be, but many other less serious will be carried out by an individual. Even when a team is reported to have carried out an investigation, it is likely that a single lead will have carried out most of the analysis. Though the scenarios were based on real incidents, they might not fully capture the complexity of actual events, limiting the generalisability. Participant recruitment was via X, (formerly Twitter), which may not represent the broader population. We did not account for other participant characteristics such as professional backgrounds of staff participants (eg, nursing, medical, etc), workplace (urban large academic centre vs rural settings, etc), health policies relevant to incident reviews in their jurisdiction or cultural backgrounds, which might influence how individuals respond to the question in this study.^22 63^ This study was not powered to detect differences in other participant grouping. Our MLMs suggested we identified a significant number of confounding factors; there are likely to be several other confounding or moderating factors that we have not explored, which impact the decisions and judgements of people investigating incidents. Identifying and exploring the impact of these factors might be of interest for future research.

## Conclusion

This study adds to the body of evidence that human cognition and bias are likely to have a significant impact on how incidents are investigated within healthcare. We highlight the conflicting views of the public, staff and experts; the difficult topic of individual responsibility, accountability and ‘blame’ and ultimately the appropriate distribution of ‘causality’ of a systems performance between individuals and systems.

## Supplementary material

10.1136/bmjqs-2024-017926 online supplemental file 1

10.1136/bmjqs-2024-017926 online supplemental file 2

10.1136/bmjqs-2024-017926 online supplemental file 3

## Data Availability

Data are available upon reasonable request.
