# Supplementary material for: Investigators are human too: outcome bias and perceptions of individual culpability in patient safety incident investigations
Source: BMJ Qual Saf. 2025 Feb 10;35(3):e017926. doi: 10.1136/bmjqs-2024-017926 (PMC13018802; doi:10.1136/bmjqs-2024-017926)
Supplement: online supplemental file 2 [file bmjqs-35-3-s002.pdf]

Scenario Study - GAR (V 10-2-22)

What's the purpose of this research?

It is estimated that 1 in 10 patients are harmed as the result of healthcare[1]; and that this is often the result of both human error and poor system design. One of the ways in which safety might be improved is to investigate these patient safety incidents (PSIs). PSIs are routinely investigated within healthcare, with the purpose of finding why the error or harm occurred. Following an investigation staff will usually identify some actions or changes that could be introduced to reduce the chance the incident is repeated or to improve safety; so called recommendations. We are interested in looking at what kind of recommendations people select.

We will present you with three fictional incidents involving a fall, delay in diagnosis and medication error. We will then ask you some questions about how we should respond to the incident and to consider some possible changes we could make to prevent it happening again. For the purposes of this study we would like you to ignore potential costs, or difficulty in putting into practice; imaging you have infinite funds and resources.

You will need to:
1) read about three incidents
2) answer some questions about how we should respond

1 Braithwaite J (2018) Changing how we think about healthcare improvement. BMJ 2018;361:k2014

Consent

Before we start we would like to check that you are happy to take part in this research study.

You are being invited to participate in a research study looking at what recommendations people select following medical errors/adverse events. This study is being done by William Lea*[1], Jane O'Hara*[1], Rebecca Lawton*[1] and Charles Vincent*[2].

We believe there are no known risks associated with this research study; however, as with any online related activity the risk of a breach is always possible. To the best of our ability your participation in this study will remain confidential, and only anonymised data will be published.

- I understand that my participation is voluntary and that I am free to withdraw at any time prior to submitting the completed questionnaire without giving any reason and without there being any negative consequences.
- I understand that my participation or my individual responses in this study will not be shared with my employer.
- I understand that my name will not be linked with the research materials, and I will not be identified or identifiable in the report or reports that result from the research.
- I agree for the data collected from me to be used in relevant future research in an anonymised form.

*[1] University of Leeds, *[2] University of Oxford

I agree with the above statements and I am happy to take part in this research:

- ○ Yes
- ○ No

Further information about your rights in relation to the information we collect can be found here: https://dataprotection.leeds.ac.uk/wp-content/uploads/sites/48/2020/11/Research-Participant-Privacy-Notice.docx

A little bit about you

How old are you?

Would you describe yourself as a

- Women
- Man
- Non-binary
- other

Prefer to self describe as

Do you work in healthcare?

- Yes
- No

A little bit about you (Non-Healthcare)

Have you ever been involved in a patient safety incident/adverse event as a:

	Yes	No	approximately how many times?	comments
Victim (patient or family member)				
None of the ways listed above (please give details in comments box)				

Have you ever had training or do you have expertise in patient safety or how to investigate adverse events? (e.g. carry out investigations, undertake relevant research)

- Yes
- No

If you have answered 'yes' please could you provide some details, such as job role/research area or content and length of training.

A little bit about you (Healthcare)

Have you ever been involved in a patient safety incident/adverse event as a:

	Yes	No	approximately how many times?	comments
Victim (patient or family member)	○	○		
Member of staff involved in the incident	○	○		
Member of the investigation team	○	○		
None of the ways listed above (please give details in comments box)	○	○		

Have you ever had training or do you have expertise in patient safety or how to investigate adverse events? (e.g. carry out investigations, undertake relevant research)

○ Yes
○ No

If you have answered 'yes' please could you provide some details, such as job role/research area or content and length of training.

What is your profession?

If you selected Other, please specify:

What is your grade? (e.g. Band 3, Foundation Year 1 doctor)

What year did you qualify?

Scenario 1

A fall on the ward: a brief summary

Doris Campbell, an 80 year old lady, was admitted to the acute medical unit with a urinary tract infection and acute confusion (delirium). Doris was assessed by the physiotherapist who noted reduced balance and the need for assistance to stand. The physiotherapist was going to pass this information onto the nurse but was asked to urgently provide chest physiotherapy to another patient who had become unwell. The nurse looking after Doris completed her falls risk assessment which suggested she was ‘high risk’; among other things this prompted that Doris should have a falls sensor(i); the nurse had a lot to do and asked if the healthcare assistant could arrange for one to be fitted. The healthcare assistant didn’t feel comfortable with how to fit a falls sensor and didn’t want to trouble the busy nurse; they decided to find someone else to help when they got a moment.

Later that day Doris was found on the floor next to her bed. She was immediately assessed by the nurse and junior doctor. Although dazed Doris had fortunately not suffered any injuries and was helped back to her bed.

Following an investigation, the following factors were thought to have contributed to this incident:

1. Doris was very confused and agitated putting her at greater risk of a fall
2. Doris was placed in a bed without a clear line of site to the staff on the ward
3. There were insufficient nursing staff on duty the day Doris was admitted
4. The physiotherapist who noted the patient’s falls risk had been distracted and so had not shared this with the nurse.
5. The nurse asked the healthcare assistant to fit the falls sensor without realising they didn’t feel confident in doing this.
6. Doris was on a number of medications before she was admitted, which could increase her falls risk. The junior doctor who initially saw Doris was not sure which of these medications to stop and so decided to leave this for the consultant review.
7. The brakes on Doris’ bed had not been applied, which moved as she had tried to stand.
8. There is no protocol for the management of confused patients.

Q1 How responsible do you feel the healthcare staff are in causing this incident?

Please don't select more than 1 answer(s) per row.

	1	2	3	4	5
1 = not at all , 5 = entirely	☐	☐	☐	☐	☐

Q2 How avoidable do you think this incident was?

Please don't select more than 1 answer(s) per row.

	1	2	3	4	5
1 = not at all, 5 = entirely	☐	☐	☐	☐	☐

Q3 How important do you think it is for an investigation to be carried out?

Please don't select more than 1 answer(s) per row.

	1	2	3	4	5
1 = not at all, 5 =very	☐	☐	☐	☐	☐

Q4 What level of harm was caused by this incident?

- ○ No/Low harm
- ○ Severe harm
- ○ Death

Recommendations

Please consider the following recommendations and select **up to five** to reduce the chance this incident happens again.

Please select no more than 5 answer(s).

- [ ] The healthcare assistant should undergo a period of supervised practice.
- [ ] Consider disciplinary process for the nurse in charge of this patients care.
- [ ] Remind all staff that brakes should be re-applied after moving a bed.
- [ ] Junior doctors to receive training on medications which increase fall’s risk.
- [ ] Introduce a checklist to ensure that confused patients receive appropriate investigations and management, including those to reduce falls risk.
- [ ] Introduce an electronic alert within the computer based prescribing system to identify those medications, which increase fall’s risk.
- [ ] New beds will be sourced which automatically apply brakes, which then have to be dis-engaged to move the bed.
- [ ] Continue the work of the Falls Reduction Committee to continuously improve falls reduction strategies such as low beds, zipper quilts, and falls action plans.
- [ ] None. I do not wish to select any recommendations

[Optional] Please include any explanation for your choices here or suggest other recommendations you might have proposed:

Scenario 2 - A missed x-ray

Incident – a brief summary

David Carter, a 72 year old man, was admitted to the acute medical unit at his local hospital. Over the last few days he had been getting some pain in his abdomen but was otherwise well. He was seen by the junior doctor (Dr A) who ordered some blood tests and an x-ray of his abdomen and chest. David's observations were normal and he looked quite well. After an hour the consultant on duty reviewed David and Dr A's plan; they agreed with the junior doctor and wrote that the x-rays would help check for any evidence of perforation in David's bowels. The consultant did not directly discuss the case with Dr A, but suggested in the written notes that a surgical review should be arranged. The x-rays were not scheduled as urgent but would be done within a few hours. Shortly before the end of Dr A's shift there was an emergency in an adjacent ward, which they assisted with. Following this emergency Dr A went to hand over, during which the doctors hand over patients and jobs to the next shift of doctors. David Carter's x-rays had not yet been completed and Dr A failed to handover these for the incoming team to chase.

Later that night David became unwell and the nursing staff asked the doctors to review him. The doctors were very concerned about David and they noticed that he had had x-rays showing a perforation in his bowel. They immediately spoke to the surgical team, and he underwent emergency surgery. The delay in diagnosis of perforation made the operation more complicated and David remained in hospital for a further week. He was discharged home and has now made a full recovery. The consultant spoke with the Dr A, who admitted that they had forgotten to hand over the task of chasing David's x-rays.

Following an investigation, the following factors were thought to have contributed to this incident:

1. David was admitted to the acute medical unit, but should have been admitted to the surgical assessment unit where staff are more familiar with perforation of the bowel.
2. David was not displaying the typical physiological signs of perforation such as low blood pressure and tachycardia, and his abdomen was soft and only mildly tender which is again, not typical of perforation.
3. Dr A was less familiar with surgical conditions.
4. The consultant did not directly discuss David's case with Dr A or handover their concerns about perforation and the need to discuss the case with the surgical team.
5. Dr A faced multiple distractions during their shift and in particular the emergency that occurred

just before handover, when Dr A reports they would usually write a list of items to handover.

6. There is no system of alerting staff when out-of-hours x-rays have been completed.
7. When x-rays are completed with obvious abnormalities there is no system of alerting the parent medical team.
8. There is no structure or standardised approach to the handover process.

Q1 How responsible do you feel the healthcare staff are in causing this incident?

Please don't select more than 1 answer(s) per row.

	1	2	3	4	5
1 = not at all , 5 = entirely	☐	☐	☐	☐	☐

Q2 How avoidable do you think this incident was?

Please don't select more than 1 answer(s) per row.

	1	2	3	4	5
1 = not at all, 5 = entirely	☐	☐	☐	☐	☐

Q3 How important do you think it is for an investigation to be carried out?

Please don't select more than 1 answer(s) per row.

	1	2	3	4	5
1 = not at all, 5 =very	☐	☐	☐	☐	☐

Q4 What level of harm was caused by this incident?

- No/Low harm
- Severe harm
- Death

Recommendations

Please consider the following recommendations and select **up to five** to reduce the chance this incident happens again.

Please select no more than 5 answer(s).

- ☐ Dr A should be suspended and further investigation into their practice carried out.
- ☐ Dr A will complete training on how to organise and prioritise tasks, including those for handover.
- ☐ Medical staff will receive training on surgical conditions, including perforation.
- ☐ A new policy for out-of-hours x-rays will be produced.
- ☐ The handover process will be standardised and a checklist developed to ensure all important information is discussed, including a prompt to handover outstanding x-rays.
- ☐ Radiographers to bleep requesting team when out-of-hours x-rays are completed to prompt review.
- ☐ All patients with abdominal pain will be admitted under the surgical team for initial assessment.
- ☐ Hire an advanced radiographer for out-of-hours to ensure that all x-rays are reviewed and abnormalities are discussed with parent medical team.
- ☐ None. I do not wish to select any recommendations

[Optional] Please include any explanation for your choices here or suggest other recommendations you might have proposed:

Scenario 3 - Wrong dose

Incident – a brief summary

Samantha Giles, a 32 year old teacher, attended her local emergency department feeling short of breath. Doctor B who assessed Samantha thought she had a blood clot in her lungs (pulmonary embolism or ‘PE’). The doctor prescribed a blood thinning injection (Dalteparin); the dose of which is calculated based on weight. There are different dose syringes based on the weight of the patient. After writing the prescription the doctor asked one of the nurses to administer the injection while they arranged a scan to confirm whether or not there was a PE.

Shortly after giving Samantha the dalteparin injection, the nurse realised they had picked up the wrong dose syringe and had in fact given her a much higher dose than intended. The nurse and doctor explained that Samantha had had a much higher dose than intended and that there was a risk of bleeding. Samantha was kept in hospital and monitored closely.

Samantha suddenly became confused and had a seizure. She became unconscious and was sent for an urgent scan of her head. The scan showed that Samantha had a large bleed inside her head. She was urgently transferred to a neurosurgery centre for treatment but unfortunately died from complications of the surgery.

Following an investigation, the following factors were thought to have contributed to this incident:

This incident was investgated and it was felt the following factors contributed to the wrong dose:

1. The emergency department did not have its full quota of nursing staff on duty, and so the nurse involved in this incident was stressed, rushed and distracted.
2. The emergency department was particularly busy with higher than usual numbers of patients.
3. The dalteparin injection comes in a variety of different dose syringes, but they all look very similar and are kept together
4. The blood thinning injection syringes are kept in a cupboard under poor lighting
5. The national 4 hour emergency department target places pressure on staff to work more quickly

Q1 How responsible do you feel the healthcare staff are in causing this incident?

Please don't select more than 1 answer(s) per row.

	1	2	3	4	5
1 = not at all , 5 = entirely	☐	☐	☐	☐	☐

Q2 How avoidable do you think this incident was?

Please don't select more than 1 answer(s) per row.

	1	2	3	4	5
1 = not at all, 5 = entirely	☐	☐	☐	☐	☐

Q3 How important do you think it is for an investigation to be carried out?

Please don't select more than 1 answer(s) per row.

	1	2	3	4	5
1 = not at all, 5 =very	☐	☐	☐	☐	☐

Q4 What level of harm was caused by this incident?

- ○ No/Low harm
- ○ Severe harm
- ○ Death

Recommendations

Please consider the following recommendations and select **up to five** to reduce the chance this incident happens again.

Please select no more than 5 answer(s).

- ☐ The nurse involved should be suspended and further investigation into their practice carried out.
- ☐ The nurse should undergo a period of supervised practice.
- ☐ Training for emergency department staff on the risks of blood thinning medications.
- ☐ Place a warning poster on the cupboard containing the blood thinning medications, to remind staff to select the correct strength syringe.
- ☐ Modify the labelling on the different dose strength blood thinning medication syringes so that they look significantly different.
- ☐ Provide emergency department staff with regular opportunities to carry out tasks under pressure in a simulated environment, with subsequent feedback/debrief.
- ☐ Switch to a different supplier of blood thinning medication syringes that have been designed specifically to reduce the risk of selecting the wrong syringe.
- ☐ Senior leadership to set up a working group and commit to a range of projects to improve patient safety in the emergency department.
- ☐ None. I do not wish to select any recommendations

[Optional] Please include any explanation for your choices here or suggest other recommendations you might have proposed:

Did you face any difficulties answering these questions?

Did you find any of the questions difficult to answer? Or do you have any other comments you would like to make?

Thank you

We are very grateful for your time in taking part in this research, and hope that the findings will improve the way in which we can learn from errors within healthcare.

If you have any questions about the research or would like to be informed about the results please contact William Lea (umwl@leeds.ac.uk)

Taking part in this survey may have raised questions for you about incidents and harm. The following organisations provide information and support:

Action Against Medical Accidents (www.avma.org.uk): whose purpose if to support people affected by avoidable harm in healthcare; to help them achieve hustice; and to promote better patient safety for all.

Harmed Patient Alliance (www.harmedpatientsalliance.org.uk): are working to provide support for patients and families following harm.

Second Victim Support (www.secondvictim.co.uk): recognises the impact that a patient safety incident has on the healthcare employee involved. Whilst the patient and their family are always the priority, this website seeks to help 'second victims' identify the types of support they may need, and signpost them towards help.

Key for selection options

2 - How old are you?

1
2
3
4
5
6
7
8
9
10
11

- 92
- 93
- 94
- 95
- 96
- 97
- 98
- 99
- 100

9 - What is your profession?

- Dietitian
- Doctor
- Healthcare Assistant
- Manager
- Midwife
- Nurse
- Occupational therapist
- Physiotherapist
- Radiographer
- Other
