# Supplementary material for: Investigators are human too: outcome bias and perceptions of individual culpability in patient safety incident investigations
Source: BMJ Qual Saf. 2025 Feb 10;35(3):e017926. doi: 10.1136/bmjqs-2024-017926 (PMC13018802; doi:10.1136/bmjqs-2024-017926)
Supplement: online supplemental file 3 [file bmjqs-35-3-s003.doc]

| **Supplemental Table 1: Full Model Results** | |  |  |  |  |  |  |
| --- | --- | --- | --- | --- | --- | --- | --- |
| **Outcome** | **Model type** | **Predictor** | **Contrast** | **Estimate [95% CI]** | **R^2^_M_ | R^2^_C_** | **ICC** | **BIC** |
| **Number of recommendations** | Model 1: adjusted for participant age + gender | Outcome | Severe vs. No/low-harm | -0.05 [-0.24, 0.13] | 1.73% | 40.33% | 39.28% | 1680 |
|  |  |  | **Death vs. No/low-harm** | **0.20** [0.02, 0.39]** |  |  |  |
|  |  | Age | - | -0.01 [-0.02, 0.00] |  |  |  |
|  |  | Gender | Male vs. Female | -0.15 [-0.46, 0.17] |  |  |  |
|  | Model 2: Model 1 variables + adjusted for scenario (1=fall, 2=x-ray, 3=drug dose) + participant group (0=public, 1=staff, 2=experts) | Outcome | Severe vs. No/low-harm | -0.07 [-0.24, 0.11] | 5.68% | 43.73% | 40.35% | 1689 |
|  |  |  | **Death vs. No/low-harm** | **0.20** [0.02, 0.38]** |  |  |  |
|  |  | Age | - | 0.00 [-0.01, 0.01] |  |  |  |
|  |  | Gender | Male vs. Female | -0.16 [-0.48, 0.16] |  |  |  |
|  |  | Scenario | **X-ray vs. Fall** | **-0.28*** [-0.46,-0.10]** |  |  |  |
|  |  |  | **Drug vs. Fall** | **-0.41*** [-0.58,-0.23]** |  |  |  |
|  |  | Participant | Staff vs. Public | 0.10 [-0.20, 0.41] |  |  |  |
|  |  | group | **Expert vs. Public** | **-0.35*[-0.71, 0.00]** |  |  |  |
|  | Model 3: Model 2 variables + participant previous incident involvement (none=0,victim=1, staff member=2, both=3) | Outcome | Severe vs. No/low-harm | -0.07 [-0.24, 0.11] | 7.38% | 44.24% | 39.79% | 1706 |
|  |  |  | **Death vs. No/low-harm** | **0.20** [0.02, 0.38]** |  |  |  |
|  |  | Age | - | 0.00 [-0.01, 0.01] |  |  |  |
|  |  | Gender | Male vs. Female | -0.13 [-0.45, 0.19] |  |  |  |
|  |  | Scenario | **X-ray vs. Fall** | **-0.28*** [-0.46,-0.10]** |  |  |  |
|  |  |  | **Drug vs. Fall** | **-0.41*** [-0.58,-0.23]** |  |  |  |
|  |  | Participant | Staff vs. Public | 0.23 [-0.15,0.61] |  |  |  |
|  |  | group | Expert vs. Public | -0.26 [-0.68,0.16] |  |  |  |
|  |  | Involvement | Victim vs. None | 0.19 [-0.15,0.54] |  |  |  |
|  |  |  | Staff member vs. None | -0.05 [-0.45, 0.35] |  |  |  |
|  |  |  | Both vs. None | -0.50~ [-1.06, 0.05] |  |  |  |
| **Recommendation Score** | Model 1: adjusted for participant age + gender | Outcome | Severe vs. No/low-harm | -0.07 [-0.15, 0.02] | 1.00% | 33.00% | 28.56% | 819 |
|  |  |  | Death vs. No/low-harm | -0.08 [-0.17, 0.00] |  |  |  |
|  |  | Age | - | 0.00 [-0.00, 0.00] |  |  |  |
|  |  | Gender | Male vs. Female | 0.03 [-0.11, 0.17] |  |  |  |
|  | Model 2: Model 1 variables + adjusted for scenario (1=fall, 2=x-ray, 3=drug dose) + participant group (0=public, 1=staff, 2=experts) | Outcome | Severe vs. No/low-harm | -0.06 [-0.15, 0.02] | 12.0% | 39.0% | 26.8% | 807 |
|  |  |  | Death vs. No/low-harm | -0.08 [-0.16, 0.00] | P=0.057 |  |  |
|  |  | Age | - | 0.00 [-0.00, 0.00] |  |  |  |
|  |  | Gender | Male vs. Female | 0.01 [-0.12, 0.14] |  |  |  |
|  |  | Scenario | **X-ray vs. Fall** | **-0.25*** [-0.33, -0.17]** |  |  |  |
|  |  |  | Drug vs. Fall | -0.05 [-0.14, 0.03] |  |  |  |
|  |  | Participant | Staff vs. Public | 0.07 [-0.05, 0.20] |  |  |  |
|  |  | group | **Expert vs. Public** | **0.37***[0.21, 0.53]** |  |  |  |
|  | Model 3: Model 2 variables + participant previous incident involvement (none=0,victim=1, staff member=2, both=3) | Outcome | Severe vs. No/low-harm | -0.06 [-0.15, 0.02] | 14.0% | 40.0% | 26.8% | 829 |
|  |  |  | Death vs. No/low-harm | -0.08 [-0.16, 0.00] |  |  |  |
|  |  | Age | - | 0.00 [-0.00, 0.00] |  |  |  |
|  |  | Gender | Male vs. Female | -0.00 [-0.13, 0.13] |  |  |  |
|  |  | Scenario | **X-ray vs. Fall** | **-0.25*** [-0.33, -0.17]** |  |  |  |
|  |  |  | Drug vs. Fall | -0.05 [-0.14, 0.03] |  |  |  |
|  |  | Participant | Staff vs. Public | -0.02 [-0.18, 0.13] |  |  |  |
|  |  | group | **Expert vs. Public** | **0.27*** [0.13, 0.40]** |  |  |  |
|  |  | Involvement | Victim vs. None | -0.08 [-0.22,0.05] |  |  |  |
|  |  |  | Staff member vs. None | 0.14 [-0.04, 0.31] |  |  |  |
|  |  |  | Both vs. None | 0.18 [-0.04, 0.41] |  |  |  |
| **Responsibility** | Model 1: adjusted for participant age + gender | Outcome | **Severe vs. No/low-harm** | **0.17** [0.02, 0.32]** | 2.55% | 52.13% | 50.87% | 1549 |
|  |  |  | **Death vs. No/low-harm** | **0.25*** [0.10, 0.41]** |  |  |  |
|  |  | Age | - | 0.00 [-0.01, 0.01] |  |  |  |
|  |  | Gender | Male vs. Female | -0.30 [-0.62, 0.01] |  |  |  |
|  | Model 2: Model 1 variables + adjusted for scenario (1=fall, 2=x-ray, 3=drug dose) + participant group (0=public, 1=staff, 2=experts) | Outcome | **Severe vs. No/low-harm** | **0.17** [0.02, 0.32]** | 12.46% | 53.67% | 47.07% | 1547 |
|  |  |  | **Death vs. No/low-harm** | **0.26*** [0.11, 0.41]** |  |  |  |
|  |  | Age | - | 0.00 [-0.01, 0.01] |  |  |  |
|  |  | Gender | Male vs. Female | -0.24 [-0.54, 0.06] |  |  |  |
|  |  | Scenario | **X-ray vs. Fall** | **-0.26*** [-0.42, -0.11]** |  |  |  |
|  |  |  | Drug vs. Fall | -0.14 [-0.29, 0.01] |  |  |  |
|  |  | Participant | Staff vs. Public | -0.28~ [-0.57, 0.01] |  |  |  |
|  |  | group | **Expert vs. Public** | **-0.90***[-1.23, -0.56]** |  |  |  |
|  | Model 3: Model 2 variables + participant previous incident involvement (none=0,victim=1, staff member=2, both=3) | Outcome | **Severe vs. No/low-harm** | **0.17** [0.02, 0.32]** | 15.51% | 54.05% | 45.62% | 1560 |
|  |  |  | **Death vs. No/low-harm** | **0.26*** [0.11, 0.41]** |  |  |  |
|  |  | Age | - | 0.00 [-0.01, 0.01] |  |  |  |
|  |  | Gender | Male vs. Female | -0.23 [-0.52, 0.06] |  |  |  |
|  |  | Scenario | **X-ray vs. Fall** | **-0.26*** [-0.42, -0.11]** |  |  |  |
|  |  |  | Drug vs. Fall | -0.14~ [-0.29, 0.01] |  |  |  |
|  |  | Participant | **Staff vs. Public** | **-0.38** [-0.72, -0.03]** |  |  |  |
|  |  | group | **Expert vs. Public** | **-1.00*** [-1.39, -0.62]** |  |  |  |
|  |  | Involvement | **Victim vs. None** | **0.52*** [0.20, 0.83]** |  |  |  |
|  |  |  | Staff member vs. None | 0.20 [-0.17, 0.57] |  |  |  |
|  |  |  | Both vs. None | 0.33 [-0.18, 0.85] |  |  |  |
| **Avoidability** | Model 1: adjusted for participant age + gender | Outcome | Severe vs. No/low-harm | -0.05 [-0.22, 0.11] | 0.93% | 32.35% | 31.71% | 1499 |
|  |  |  | Death vs. No/low-harm | -0.04 [-0.21, 0.12] |  |  |  |
|  |  | Age | - | 0.00 [0.00, 0.01] |  |  |  |
|  |  | Gender | Male vs. Female | -0.16 [-0.41, 0.09] |  |  |  |
|  | Model 2: Model 1 variables + adjusted for scenario (1=fall, 2=x-ray, 3=drug dose) + participant group (0=public, 1=staff, 2=experts) | Outcome | Severe vs. No/low-harm | -0.04 [-0.19, 0.11] | 10.46% | 39.70% | 32.66% | 1477 |
|  |  |  | Death vs. No/low-harm | -0.03 [-0.19, 0.12] |  |  |  |
|  |  | Age | - | 0.00 [-0.01, 0.01] |  |  |  |
|  |  | Gender | Male vs. Female | -0.09 [-0.33, 0.16] |  |  |  |
|  |  | Scenario | X-ray vs. Fall | 0.04 [-0.11, 0.20] |  |  |  |
|  |  |  | **Drug vs. Fall** | **0.47*** [0.31, 0.62]** |  |  |  |
|  |  | Participant | **Staff vs. Public** | **-0.37*** [-0.61, -0.13]** |  |  |  |
|  |  | group | **Expert vs. Public** | **-0.49***[-0.76, -0.22]** |  |  |  |
|  | Model 3: Model 2 variables + participant previous incident involvement (none=0,victim=1, staff member=2, both=3) | Outcome | Severe vs. No/low-harm | -0.04 [-0.19, 0.11] | 11.24% | 40.21% | 32.64 | 1498 |
|  |  |  | Death vs. No/low-harm | -0.03 [-0.19, 0.12] |  |  |  |
|  |  | Age | - | 0.00 [-0.01, 0.01] |  |  |  |
|  |  | Gender | Male vs. Female | -0.07 [-0.31, 0.18] |  |  |  |
|  |  | Scenario | X-ray vs. Fall | 0.04 [-0.11, 0.20] |  |  |  |
|  |  |  | **Drug vs. Fall** | **0.47*** [0.31, 0.62]** |  |  |  |
|  |  | Participant | Staff vs. Public | -0.25~ [-0.55, 0.04] |  |  |  |
|  |  | group | **Expert vs. Public** | **-0.38** [-0.70, -0.05]** |  |  |  |
|  |  | Involvement | Victim vs. None | 0.11 [-0.15, 0.38] |  |  |  |
|  |  |  | Staff member vs. None | -0.19 [-0.50, 0.12] |  |  |  |
|  |  |  | Both vs. None | -0.15 [-0.58, 0.28] |  |  |  |
| **Importance of Investigating** | Model 1: adjusted for participant age + gender | Outcome | **Severe vs. No/low-harm** | **0.38*** [0.24, 0.51]** | 10.46% | 47.89% | 41.81% | 1345 |
|  |  |  | **Death vs. No/low-harm** | **0.63*** [0.50, 0.76]** |  |  |  |
|  |  | Age | **-** | **0.01** [0.00, 0.02]** |  |  |  |
|  |  | Gender | Male vs. Female | 0.06 [-0.18, 0.30] |  |  |  |
|  | Model 2: Model 1 variables + adjusted for scenario (1=fall, 2=x-ray, 3=drug dose) + participant group (0=public, 1=staff, 2=experts) | Outcome | **Severe vs. No/low-harm** | **0.39*** [0.26, 0.51]** | 13.30% \| 51.93% | 44.55% | 1353 |
|  |  |  | **Death vs. No/low-harm** | **0.64*** [0.51, 0.77]** |  |  |  |
|  |  | Age | **-** | **0.01** [0.00, 0.02]** |  |  |  |
|  |  | Gender | Male vs. Female | 0.04 [-0.20, 0.28] |  |  |  |
|  |  | Scenario | X-ray vs. Fall | -0.11 [-0.23, 0.02] |  |  |  |
|  |  |  | **Drug vs. Fall** | **0.24*** [0.11, 0.37]** |  |  |  |
|  |  | Participant | Staff vs. Public | 0.11 [-0.13, 0.34] |  |  |  |
|  |  | group | Expert vs. Public | -0.05[-0.32, 0.22] |  |  |  |
|  | Model 3: Model 2 variables + participant previous incident involvement (none=0,victim=1, staff member=2, both=3) | Outcome | **Severe vs. No/low-harm** | **0.38*** [0.26, 0.51]** | 14.97% \| 52.35% | 43.96% | 1371 |
|  |  |  | **Death vs. No/low-harm** | **0.64*** [0.51, 0.77]** |  |  |  |
|  |  | Age | - | **0.01** [0.00, 0.02]** |  |  |  |
|  |  | Gender | Male vs. Female | 0.06 [-0.18, 0.30] |  |  |  |
|  |  | Scenario | X-ray vs. Fall | -0.11 [-0.23, 0.02] |  |  |  |
|  |  |  | **Drug vs. Fall** | **0.24*** [0.11, 0.37]** |  |  |  |
|  |  | Participant | Staff vs. Public | 0.11 [-0.18, 0.40] |  |  |  |
|  |  | group | Expert vs. Public | -0.07 [-0.38, 0.25] |  |  |  |
|  |  | Involvement | **Victim vs. None** | **0.31** [0.04, 0.57]** |  |  |  |
|  |  |  | Staff member vs. None | 0.08 [-0.23, 0.39] |  |  |  |
|  |  |  | Both vs. None | -0.06 [-0.48, 0.36] |  |  |  |

***Supplemental Table 2: Differences between Participant Groups***

| **Comparison** | **Responsibility** | | | | **Avoidability** | | | | **Number of Recommendations** | | | | **Recommendation Score** | | | |
| --- | --- | --- | --- | --- | --- | --- | --- | --- | --- | --- | --- | --- | --- | --- | --- | --- |
|  | **Difference** | **95% CI-** | **95% CI+** | **p** | **Difference** | **95% CI-** | **95% CI+** | **p** | **Difference** | **95% CI-** | **95% CI+** | **p** | **Difference** | **95% CI-** | **95% CI+** | **p** |
| Public vs Staff | 0.28 | -0.01 | 0.57 | 0.056 | **0.37** | 0.13 | 0.61 | 0.002 | -0.10 | -0.41 | 0.21 | 0.51 | -0.05 | -0.15 | 0.05 | 0.338 |
| Public vs Clinical Experts | **0.85** | 0.48 | 1.22 | <0.001 | **0.53** | 0.22 | 0.84 | < .001 | 0.38 | -0.01 | 0.77 | 0.06 | **-0.32** | -0.46 | -0.18 | < .001 |
| Public vs Non-Clinical Experts | **1.01** | 0.44 | 1.58 | <0.001 | 0.38 | -0.09 | 0.85 | 0.108 | 0.29 | -0.32 | 0.90 | 0.35 | **-0.35** | -0.55 | -0.15 | < .001 |
| Staff vs Clinical Experts | **0.57** | 0.18 | 0.96 | 0.006 | 0.16 | -0.17 | 0.49 | 0.335 | **0.48** | 0.05 | 0.91 | 0.03 | **-0.27** | -0.41 | -0.13 | < .001 |
| Staff vs Non-Clinical Experts | **0.73** | 0.14 | 1.32 | 0.015 | 0.01 | -0.46 | 0.48 | 0.973 | 0.39 | -0.24 | 1.02 | 0.22 | **-0.30** | -0.50 | -0.10 | 0.004 |
| Clinical Experts vs Non-Clinical Experts | 0.16 | -0.47 | 0.79 | 0.614 | -0.15 | -0.66 | 0.36 | 0.560 | -0.09 | -0.76 | 0.58 | 0.80 | -0.03 | -0.25 | 0.19 | 0.763 |
| Means that were produced to calculate between mean differences were adjusted for age, gender, scenario, and outcome. | | | | | | | | | | | | | | | | |
